# Dietary Polyphenol Acteoside‐Related Molecular Signatures in Clear Cell Renal Cell Carcinoma: Multi‐Omics Profiling and Functional Validation of IMPDH1

**DOI:** 10.1002/fsn3.72340

**Published:** 2026-09-02

**Authors:** Xu Chen, Dong Zhang, Ziyu Ji, Shuxian Sun, Jia Wang, Shuijie Shen, Jian Zhu

**Affiliations:** ^1^ Nantong Hospital of Traditional Chinese Medicine Clinical Innovation Research Center, Affiliated Traditional Chinese Medicine Hospital of Nantong University Nantong University Nantong China; ^2^ Clinical Medical Research Center, Nantong Hospital of Traditional Chinese Medicine, Affiliated Traditional Chinese Medicine Hospital of Nantong University Nantong University Nantong China

**Keywords:** acteoside, clear cell renal cell carcinoma, IMPDH1, molecular subtype, multi‐omics

## Abstract

Clear cell renal cell carcinoma (ccRCC) is characterized by substantial metabolic and molecular heterogeneity, but the disease‐relevant programs associated with acteoside, a dietary polyphenol, remain poorly understood. We integrated predicted acteoside targets with bulk, single‐cell, and spatial transcriptomic data from ccRCC and combined molecular subtyping with cross‐cohort machine‐learning analysis. Acteoside‐related signatures were preferentially enriched in malignant compartments and increased with tumor grade and stage. Consensus clustering identified two molecular subtypes with distinct biological and clinical features. C1 was associated with immune activation, metabolic activity, and more favorable survival, whereas C2 showed greater genomic instability, reduced renal epithelial differentiation, and poorer outcomes. We further benchmarked multiple machine‐learning strategies and established a 10‐gene prognostic model that retained predictive performance across independent cohorts, with IMPDH1 emerging as the strongest risk‐associated feature. Functional experiments confirmed the biological relevance of IMPDH1: its knockdown suppressed ccRCC cell proliferation, DNA synthesis, colony formation, and migration, whereas overexpression produced the opposite effects. Together, these findings indicate that acteoside‐related molecular signatures capture clinically relevant heterogeneity in ccRCC and provide a framework for linking dietary‐polyphenol‐related molecular space with tumor biology. The identification and functional validation of IMPDH1 further highlight its potential importance in ccRCC progression.

## Graphical abstract

Acteoside‐related molecular signatures were integrated with bulk, single‐cell, and spatial transcriptomic data to characterize clear cell renal cell carcinoma (ccRCC) heterogeneity. Two molecular subtypes (C1 and C2) showed distinct clinical, metabolic, immune, and genomic features. Cross‐cohort machine learning yielded a 10‐gene prognostic signature, with IMPDH1 emerging as the leading risk‐associated feature. Functional assays showed that IMPDH1 knockdown suppressed proliferation, DNA synthesis, colony formation, and migration, whereas overexpression enhanced these phenotypes.

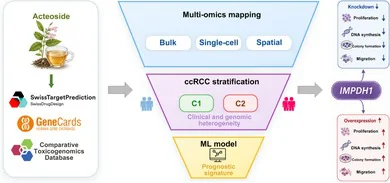

## 1 Introduction

Clear cell renal cell carcinoma (ccRCC) makes up roughly 75% of kidney cancer diagnoses and is the most common RCC subtype (Siegel et al. 2026; Young et al. 2024). Surgery, targeted agents, and immunotherapy have expanded the options for advanced disease, yet many patients still relapse or develop resistance (Cotta et al. 2023; Chen et al. 2023). A central feature of ccRCC is its altered metabolism: glucose and lipid pathways are rewired in ways that fuel tumor growth and dampen local immune control (Burgers et al. 2025; Zhang, Chen, et al. 2026). The genomic landscape adds another layer of complexity—VHL, PBRM1, and BAP1 are frequently mutated, and the particular combination of alterations shapes both intratumoral heterogeneity and clinical trajectory (Ricketts et al. 2018). Markers that can read metabolic state alongside genomic context and modifiable exposures may point to vulnerabilities worth pursuing for risk assessment.

Acteoside, also known as verbascoside, is a phenylethanoid glycoside and dietary polyphenol found in several medicinal and edible plants (Mohammed et al. 2025; Zhang, Jia, et al. 2026). Importantly, its relevance to food and nutrition is not merely conceptual. Verbascoside has been identified and quantified in edible table olives and is also a major phenylpropanoid component of aqueous lemon verbena preparations consumed as herbal infusions (Pereira et al. 2006; Bilia et al. 2008). Acteoside has additionally been described as a principal bioactive component of Cistanche‐derived tea used in functional beverages (Cui et al. 2018). These food and beverage matrices place acteoside within the broader field of dietary polyphenols, for which nutritional effects depend not only on intrinsic biological activity but also on the food matrix, gastrointestinal stability, absorption, biotransformation, and the concentrations ultimately reaching target tissues. These considerations are particularly important for acteoside because exposure to the intact parent compound after oral intake appears to be limited. Simulated gastrointestinal digestion and intestinal epithelial models have shown that acteoside is only partially bioaccessible and exhibits low cellular uptake, while experimental pharmacokinetic studies likewise indicate low systemic bioavailability of intact acteoside after oral administration (Cardinali et al. 2011; Zhou et al. 2018). In parallel, acteoside undergoes extensive gastrointestinal and microbial transformation. Human intestinal bacteria can convert acteoside through deglycosylation, de‐rhamnosylation, and related pathways into smaller phenolic metabolites, including hydroxytyrosol and 3‐hydroxyphenylpropionic acid, while additional dietary‐acteoside studies have identified caffeic acid among its metabolites (Li et al. 2016). Some of these metabolites retain biological activity. Therefore, the nutritional consequences of consuming acteoside‐containing foods may reflect the combined effects of the parent molecule, gastrointestinally generated metabolites, and the food matrix rather than systemic exposure to unchanged acteoside alone.

Dietary polyphenols are increasingly studied in relation to the prevention of non‐communicable chronic diseases because they can influence oxidative stress, inflammation, metabolic homeostasis, and cell‐survival signaling. In experimental cancer models, acteoside has been reported to affect proliferation, apoptosis, and epithelial‐to‐mesenchymal transition (EMT) (Jiang et al. 2024; Khan et al. 2022). However, pharmacological activity observed in experimental systems should not be equated directly with effects achievable through dietary intake, particularly for compounds with limited oral bioavailability. A key unresolved question is therefore whether disease‐relevant host molecular programs can be identified within the predicted molecular space associated with acteoside and subsequently used as testable endpoints in nutrition‐oriented studies (Zhou et al. 2024).

Multi‐omics analyses have improved the study of tumor biology by linking genomic, transcriptomic, and cellular heterogeneity (Zhang, Li, et al. 2026; Zhang et al. 2025). In ccRCC, large‐scale datasets have enabled molecular subtyping with distinct clinical and biological features, supporting transcriptome‐based risk stratification. Single‐cell RNA sequencing and spatial transcriptomics now provide higher‐resolution views of tumor heterogeneity and the tumor microenvironment, including cellular interactions and spatial organization within tumor tissues (Obradovic et al. 2021). However, many tumor‐omics studies remain primarily data‐driven and do not incorporate food‐derived bioactive compounds as an a priori biological framework. Conversely, studies of dietary polyphenols frequently lack cellular and spatial resolution in diseased human tissues. Integrating these two perspectives may therefore help bridge food‐derived bioactive research with disease‐relevant molecular biology without assuming a direct preventive effect.

Here, we investigated the clinical and biological significance of acteoside‐related molecular signatures in ccRCC. Using an integrated framework combining bulk, single‐cell, and spatial transcriptomics, we examined where these target‐associated signatures were expressed within the tumor microenvironment and how they related to patient outcomes, metabolic programs, tumor stemness, and genomic instability. We then constructed a cross‐cohort prognostic model to prioritize disease‐relevant molecular nodes within this acteoside‐related target space and experimentally evaluated the function of IMPDH1 in ccRCC cells. Importantly, this design was not intended to demonstrate that dietary acteoside prevents ccRCC or that acteoside directly targets IMPDH1. Instead, it was designed to provide a molecular framework linking dietary‐polyphenol‐related biology with ccRCC heterogeneity and to identify candidate pathways and biomarkers for subsequent studies incorporating food source, bioavailability, gastrointestinal metabolism, and physiologically relevant dietary exposure.

## 2 Methods

### 2.1 Integration of Acteoside‐Related Gene Sets and Clinicopathological Analysis

We started from three resources that collect pharmacological and toxicogenomic information: the Comparative Toxicogenomics Database (CTD), GeneCards, and SwissTargetPrediction (Davis et al. 2023; Daina et al. 2019). From CTD, we retained targets with more than one reported interaction; from GeneCards, those scoring above 1.0; from SwissTargetPrediction, any entry with a nonzero probability. Across the three sources, this gave 22, 22, and 15 genes, respectively. Because the overall yield was modest, we kept every gene that passed its database‐specific filter rather than forcing an intersection. Symbols were standardized to HGNC nomenclature before merging, and the per‐database gene lists appear in Table S1. Transcriptomic and clinical data for ccRCC came from The Cancer Genome Atlas (TCGA). Single‐sample Gene Set Enrichment Analysis (ssGSEA) produced three per‐patient scores—ActeosideCTD, ActeosideGC, and ActeosideSTP—each reflecting the aggregate expression of the targets from the corresponding database. We then asked whether these scores tracked with disease severity by comparing them across normal versus tumor tissue, Grade 1–2 versus Grade 3–4, and Stage I–II versus Stage III–IV.

### 2.2 Single‐Cell Transcriptomic Landscape Characterization

scRNA‐seq profiles from 12 samples (7 ccRCC and 5 normal tissues) were obtained from GSE156632 and analyzed with Seurat (v5.0.3) (Zhang et al. 2022). Cells with fewer than 300 genes, > 10% mitochondrial transcripts, or > 3% hemoglobin transcripts were excluded. After normalization and scaling, Harmony was used to integrate datasets and reduce batch effects. PCA and UMAP were applied for dimensionality reduction, followed by clustering at a resolution of 0.2. Cell identities were assigned according to established marker genes, and AddModuleScore was used to quantify enrichment of the three acteoside‐associated gene signatures at the single‐cell level. In the present study, malignant cells were analyzed as a major compartment to compare malignant versus non‐malignant enrichment of acteoside‐related signatures; we did not further subdivide malignant cells into intratumoral subclones because the primary objective was compartment‐level localization rather than malignant‐cell‐state taxonomy.

### 2.3 Spatial Transcriptomic Architecture Analysis

Spatial transcriptomic data (GSM5924035 and GSM5924036) were processed with the SpaCET pipeline (Meylan et al. 2022). After SCTransform normalization, we ran spatial clustering and mapped the histological layout. SpaCET‐based deconvolution estimated the cell‐type composition around the tumor‐stroma boundary, and each capture spot was then called as malignant (Mal) or non‐malignant (nMal). Using this spot‐level annotation, we examined whether acteoside‐related module scores were spatially biased toward the malignant compartment. With only two sections available, these data serve as supportive spatial evidence rather than definitive population‐level validation.

### 2.4 Acteoside‐Related Molecular Subtypes

We used the ConsensusClusterPlus R package to explore whether acteoside‐associated genes could separate ccRCC samples into distinct transcriptional groups. Clustering was run with hierarchical agglomerative clustering and Spearman distance, using 1000 resampling iterations, a subsampling fraction of 0.8 on items, and all features retained (pFeature = 1.0), with a fixed seed of 2020103. Solutions from *K* = 2 through *K* = 8 were compared on Silhouette Score, Dunn Index, Calinski‐Harabasz Index, and cluster‐size stability to pick the optimal *K*. We then examined cluster separation and between‐cluster correlation through consensus matrix heatmaps and CDF plots.

### 2.5 Survival and Prognostic Analysis

We compared overall survival (OS), disease‐specific survival (DSS), and progression‐free interval (PFI) between the two molecular subtypes. Survival curves were estimated by the Kaplan–Meier method and compared with the log‐rank test; results are reported as hazard ratios (HRs) with 95% confidence intervals (CIs). Each gene in the acteoside‐related modules went through univariate Cox regression, and those reaching *p* < 0.05 were taken forward. To test whether the final risk score carried prognostic weight beyond standard clinical variables, we ran multivariate Cox models that included age, sex, pathological grade, pathological stage, T stage, M stage, and the risk score itself.

### 2.6 Functional and Pathway Enrichment Analysis

Gene Set Variation Analysis (GSVA) with hallmark gene sets was used to compare pathway activity between the two subtypes. For each cluster, we took the 100 most differentially expressed genes and ran Gene Ontology (GO) and KEGG enrichment through clusterProfiler. The output was displayed as GSVA score distributions, *Z*‐score bubble plots across BP, CC, and MF categories, and chord diagrams linking genes to enriched pathways. We note that negative *Z*‐scores reflect relative underrepresentation of a term in one group compared with the other; they do not, by themselves, establish anatomical or structural loss.

### 2.7 Genome Instability and Tumor Stemness Analysis

To characterize the genomic landscape of the subtypes, somatic copy number alteration (SCNA) data were used to calculate amplification and deletion frequencies across chromosome arms. Genomic burden was quantified using Broad Gain/Loss Load and Focal Gain/Loss Load, followed by comparisons between C1 and C2. We also evaluated correlations between acteoside‐related scores (CTD, GC, and STP) and tumor stemness. The stemness indices included mRNAsi (transcriptome‐derived), mDNAsi (DNA methylation‐derived), and EREG‐mRNAsi/mDNAsi scores, which were analyzed using Pearson correlation.

### 2.8 Somatic Mutation and Tumor Mutational Burden (TMB) Analysis

Somatic alteration profiles for the KIRC cohort were analyzed with the maftools R package. Tumor mutational burden (TMB) was defined as the total number of non‐synonymous variants per megabase. The Wilcoxon rank‐sum test was used to compare TMB levels between the C1 and C2 subtypes. Pearson correlation analysis was used to examine relationships between acteoside‐associated scores and both synonymous and non‐synonymous mutation counts. Subtype‐specific genomic drivers were identified by differential mutation analysis based on odds ratios (ORs) for high‐frequency genes. Co‐occurrence and mutual exclusivity were tested using pairwise Fisher's exact tests. Associations between key genetic alterations and clinical progression were visualized using ternary plots across stages and grades.

### 2.9 Construction and Validation of the Integrated Machine Learning Prognostic Signature

Genes showing prognostic relevance in univariate Cox analysis were entered into a combined machine‐learning pipeline for model development. TCGA‐KIRC served as the derivation cohort, whereas ICGC‐RECA‐EU and E‐MTAB‐1980 served as independent validation cohorts. Before model fitting, expression data were restricted to genes shared by all three cohorts and then normalized. The model with the highest mean C‐index across all cohorts was selected as the optimal model. In the selected StepCox [both] + RSF framework, StepCox first screened candidate variables, and RSF was then fitted using log‐rank splitting, node size = 5, and a fixed random seed of 1234. After an initial run of 1000 trees, the optimal number of trees was selected according to the lowest out‐of‐bag error. Patients were divided into high‐ and low‐risk groups using the median risk score. Time‐dependent ROC curves and Kaplan–Meier curves were used to assess 1‐, 3‐, and 5‐year overall survival prediction. Calibration plots, decision curve analysis (DCA), and nomogram construction were used to further assess predictive consistency and possible clinical net benefit in the TCGA, ICGC‐RECA‐EU, and E‐MTAB‐1980 cohorts. Finally, SHAP (SHapley Additive exPlanations) analysis was used to determine the contribution of each predictor to the final risk score.

### 2.10 Cell Lines and Cell Culture

Human renal cell carcinoma cell lines 786‐O, 769‐P, Caki‐1, A498, and OS‐RC‐2, together with the normal renal tubular epithelial cell line HK‐2, were used in this study. Based on the expression patterns, 786‐O and 769‐P cells were selected for knockdown experiments, and Caki‐1 cells were used for IMPDH1 overexpression. Cells were cultured in RPMI‐1640 medium containing 10% fetal bovine serum (FBS) and 1% penicillin–streptomycin at 37°C in a humidified incubator with 5% CO_2_.

### 2.11 Cell Transfection

To modulate IMPDH1 expression, Lipofectamine 3000 was utilized to introduce either of two targeted siRNAs (siIMPDH1#1, siIMPDH1#2) or a scrambled sequence (siControl) into 786‐O and 769‐P cells. In parallel, Caki‐1 cells were transfected with an IMPDH1 overexpression construct (oe‐IMPDH1) or a corresponding empty vector. Cells were collected 48 h post‐treatment for downstream phenotypic evaluations and to verify transfection efficiency.

### 2.12 Proliferation and Clonogenic Assays

Cellular viability and growth dynamics were monitored employing the CCK‐8 assay. Transfectants were seeded into 96‐well plates at a density of 2 × 10^3^ cells per well. At 24‐h intervals (ranging from 0 to 72 h), the cultures were incubated with 10 μL of CCK‐8 solution for 1 to 2 h, after which the absorbance was recorded at 450 nm to plot proliferation curves. For clonogenic assessment, 1000 cells were plated per well in 6‐well dishes and cultured for 10 to 14 days without disturbance. The resulting macroscopic colonies were fixed with 4% paraformaldehyde and stained using a 0.1% crystal violet solution. Active DNA replication was further quantified via an EdU incorporation assay. Cells in 24‐well plates were incubated with EdU reagent for 2 h at 37°C, followed by formaldehyde fixation and permeabilization using 0.3% Triton X‐100. Nuclei were counterstained with DAPI, allowing EdU‐positive proliferating cells to be visually scored under a fluorescence microscope.

### 2.13 Transwell Migration Assay

Migration experiments were conducted in modified Boyden chambers equipped with 8 μm porous membranes. Briefly, 2 × 10^4^ cells resuspended in serum‐free medium were seeded into the apical chambers. The basal compartments were filled with medium enriched with 10% FBS to establish a chemotactic gradient. After incubating for 24 h at 37°C, cells remaining on the upper membrane surface were mechanically removed with a cotton swab. Cells that successfully migrated to the basolateral surface were fixed, visualized with crystal violet stain, and subsequently documented via inverted microscopy.

### 2.14 Statistical Analysis

Data are shown as mean ± SD from three or more independent biological replicates, unless noted otherwise. Group comparisons used two‐tailed Student's *t*‐tests when distributions were roughly normal, and Wilcoxon rank‐sum tests otherwise; three or more groups were compared by one‐way ANOVA or the Kruskal‐Wallis test, depending on the data. Continuous‐variable associations were assessed by Pearson or Spearman correlation as appropriate. *p* values from enrichment or multiple‐testing procedures were corrected by the Benjamini‐Hochberg method where applicable. Survival was analyzed by Kaplan–Meier curves with log‐rank tests, and prognostic associations were modeled by Cox proportional hazards regression. Significance was set at *p* < 0.05 (two‐sided).

## 3 Results

### 3.1 Acteoside Signatures Are Enriched in Malignant Cells and Advanced Tumors

**FIGURE 1:**
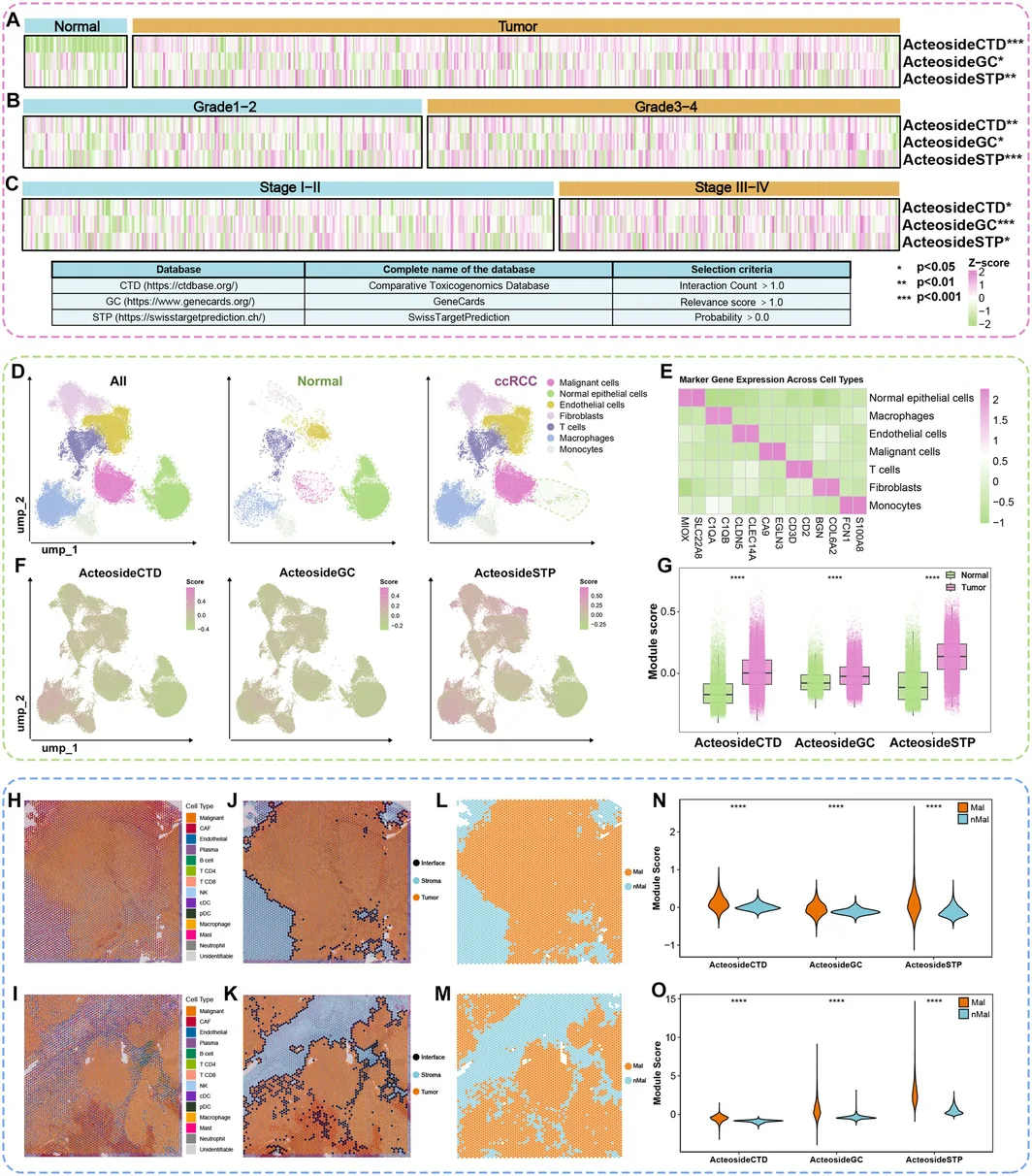
Multi‐omic characterization of acteoside‐related gene signatures in ccRCC. (A–C) Heatmaps and clinical correlation analyses showing ssGSEA scores for three acteoside‐related modules across tissue type, pathological grade, and clinical stage: (A) Normal versus tumor tissues, (B) Grade 1–2 versus Grade 3–4, and (C) Stage I–II versus Stage III–IV. (D) UMAP visualization of integrated scRNA‐seq data from GSE156632, showing major cell types in all samples, normal tissues, and ccRCC tissues. (E) Dot plot showing canonical marker‐gene expression across annotated cell lineages. (F) UMAP plots showing module scores of the three acteoside‐related signatures in the single‐cell landscape. (G) Box plots comparing module scores between normal epithelial cells and malignant cells. (H–M) Spatial transcriptomic analysis of two representative ccRCC samples (GSM5924035 and GSM5924036): (H, I) Spatial mapping of cell types; (J, K) Histological domains including tumor, stroma, and interface regions; and (L, M) Spatial distribution of malignant (Mal) and non‐malignant (nMal) spots. (N, O) Violin plots comparing acteoside‐related module scores between Mal and nMal spots in both spatial transcriptomic samples.

All three acteoside‐related module scores—ActeosideCTD, ActeosideGC, and ActeosideSTP—were higher in tumors than in adjacent normal kidney (Figure 1A), and they rose stepwise from low‐grade to high‐grade disease and from early to late stage (Figure 1B,C). In the GSE156632 single‐cell data, UMAP projection separated the expected major lineages: malignant cells along with distinct stromal and immune populations (Figure 1D). Canonical marker expression supported the accuracy of cell‐type annotation in normal and ccRCC samples (Figure 1E). Module‐score projection showed that acteoside‐related signatures were enriched mainly in malignant cell clusters rather than in normal epithelial cells or stromal populations (Figure 1F,G). Spatial transcriptomic analysis of two ccRCC sections further mapped these signatures within tissue architecture (Figure 1H–M). In both samples (GSM5924035 and GSM5924036), malignant (Mal) spots had higher module scores than non‐malignant (nMal) spots, including regions at the tumor‐stroma interface (Figure 1N,O). These single‐cell and spatial analyses were therefore interpreted as evidence that acteoside‐related signatures preferentially localized to malignant compartments, rather than as a fine‐grained classification of malignant‐cell sub‐states.

### 3.2 Acteoside‐Related Molecular Subtypes Define Distinct Prognostic Patterns in ccRCC

**FIGURE 2:**
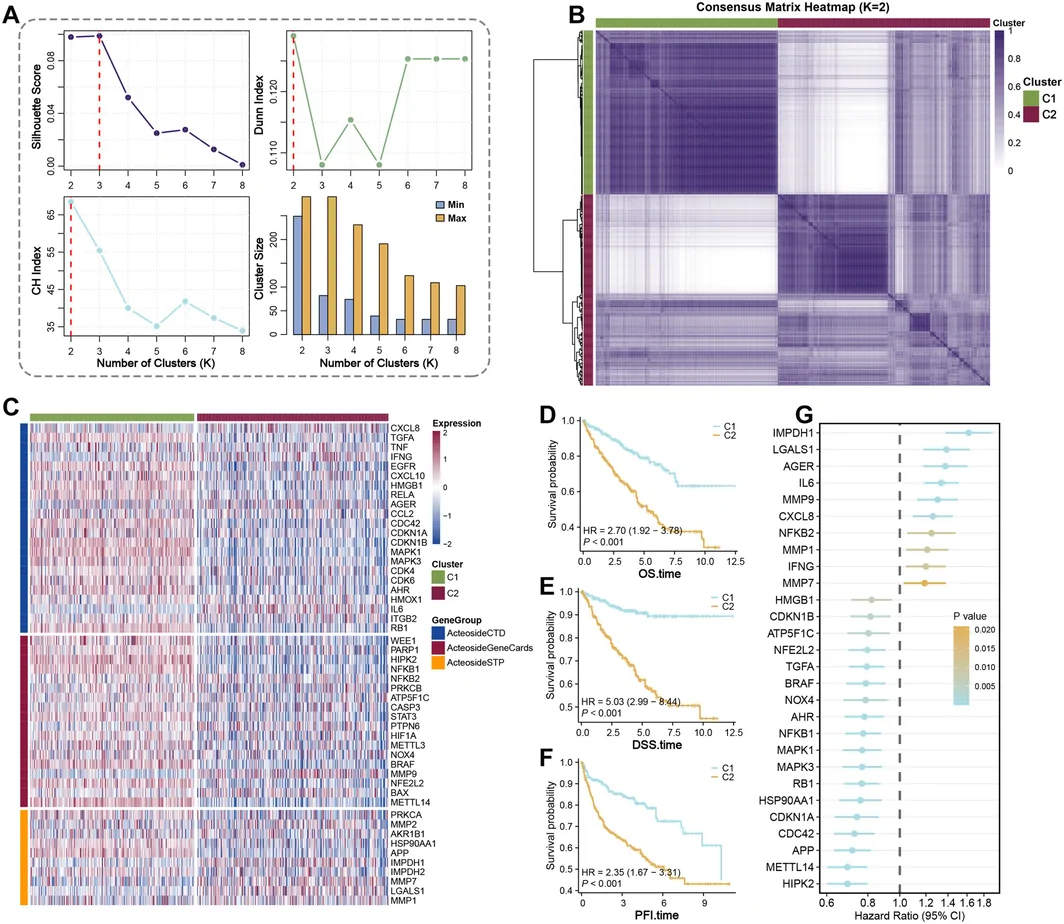
Acteoside‐related molecular subtypes are associated with distinct expression patterns and clinical outcomes in ccRCC. (A) Clustering performance evaluated by silhouette score, Dunn index, Calinski‐Harabasz (CH) index, and cluster size across different *K* values. (B) Consensus matrix heatmap showing sample‐clustering stability at *K* = 2. (C) Heatmap showing acteoside‐related gene‐expression patterns in the two molecular subtypes. (D–F) Kaplan–Meier curves comparing overall survival (OS), disease‐specific survival (DSS), and progression‐free interval (PFI) between C1 and C2. (G) Forest plot of univariate Cox regression results for acteoside‐related genes in ccRCC. CI, confidence interval; HR, hazard ratio.

To delineate transcriptional heterogeneity related to acteoside‐associated genes, we performed unsupervised clustering and identified two stable molecular subtypes, C1 and C2. *K* = 2 showed the best overall clustering performance and clear separation between subgroups (Figure 2A,B). The C1 and C2 groups included 249 and 290 patients, respectively. Heatmap analysis showed distinct expression patterns of acteoside‐related genes derived from CTD, GeneCards, and STP, indicating differences in the biological programs associated with acteoside (Figure 2C). Survival analysis showed that patients in the C2 subtype had worse OS, DSS, and PFI than patients in the C1 subtype, suggesting that this molecular classification is clinically relevant in ccRCC (Figure 2D–F). Univariate Cox regression further identified multiple acteoside‐related genes associated with prognosis, including both risk‐associated and protective genes (Figure 2G).

### 3.3 Functional Characterization and Biological Processes of Acteoside‐Related Subtypes

**FIGURE 3:**
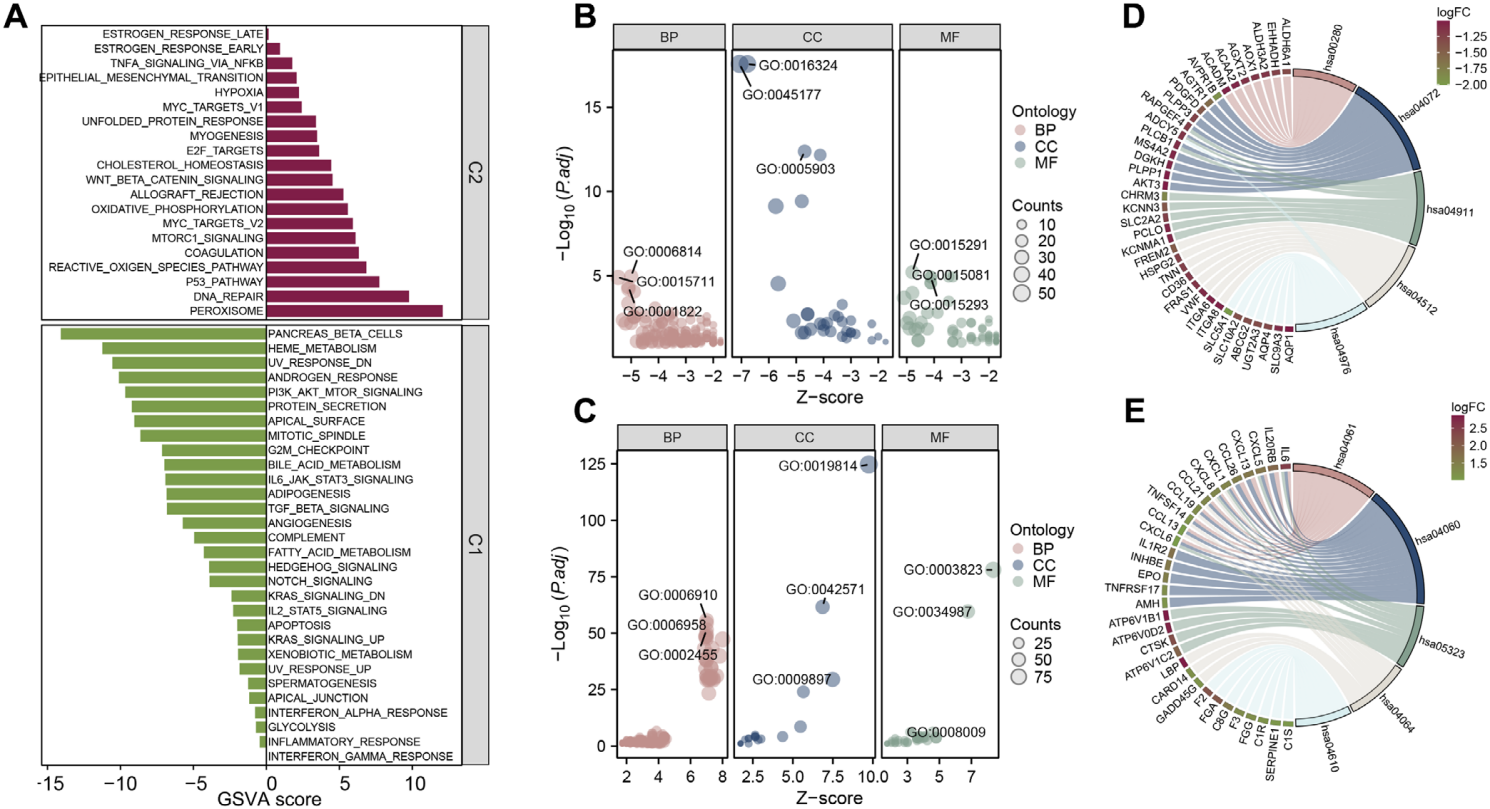
Biological and metabolic characterization of acteoside‐associated molecular subtypes. (A) Hallmark GSVA scores in C1 and C2. (B, C) GO enrichment of C2‐ (B) and C1‐associated genes (C). *Z*‐scores indicate relative enrichment across BP, CC, and MF terms. (D, E) Chord diagrams showing subtype‐associated genes and enriched KEGG pathways.

To characterize biological differences between the two subtypes, we performed GSVA and GO/KEGG enrichment analyses. Hallmark GSVA showed that C1 was enriched for immune‐active and metabolic pathways, including interferon‐gamma response, inflammatory response, and glycolysis, whereas C2 showed activation of DNA repair and the p53 pathway (Figure 3A). GO analysis further separated the two groups: C2‐associated genes showed relatively lower enrichment scores for terms related to normal renal epithelial features, including apical plasma membrane (GO:0016324) and brush border (GO:0005903), as reflected by negative *Z*‐scores (Figure 3B). In contrast, C1 showed enrichment of immunoglobulin complex (GO:0019814) and circulating immunoglobulin complex (GO:0042571), with positive *Z*‐scores (Figure 3C). KEGG chord diagrams indicated that C1‐related signatures were mainly involved in physiological transport and metabolic processes (Figure 3D), whereas C2‐specific genes mapped to cytokine‐cytokine receptor interaction (hsa04060) and inflammatory pathways (Figure 3E). Together, these findings suggest that C1 retains a more immune‐active and metabolically organized profile, whereas C2 is associated with reduced renal epithelial differentiation signatures and altered inflammatory signaling.

### 3.4 Genomic Instability and Tumor Stemness Characteristics of Acteoside‐Related Subtypes

**FIGURE 4:**
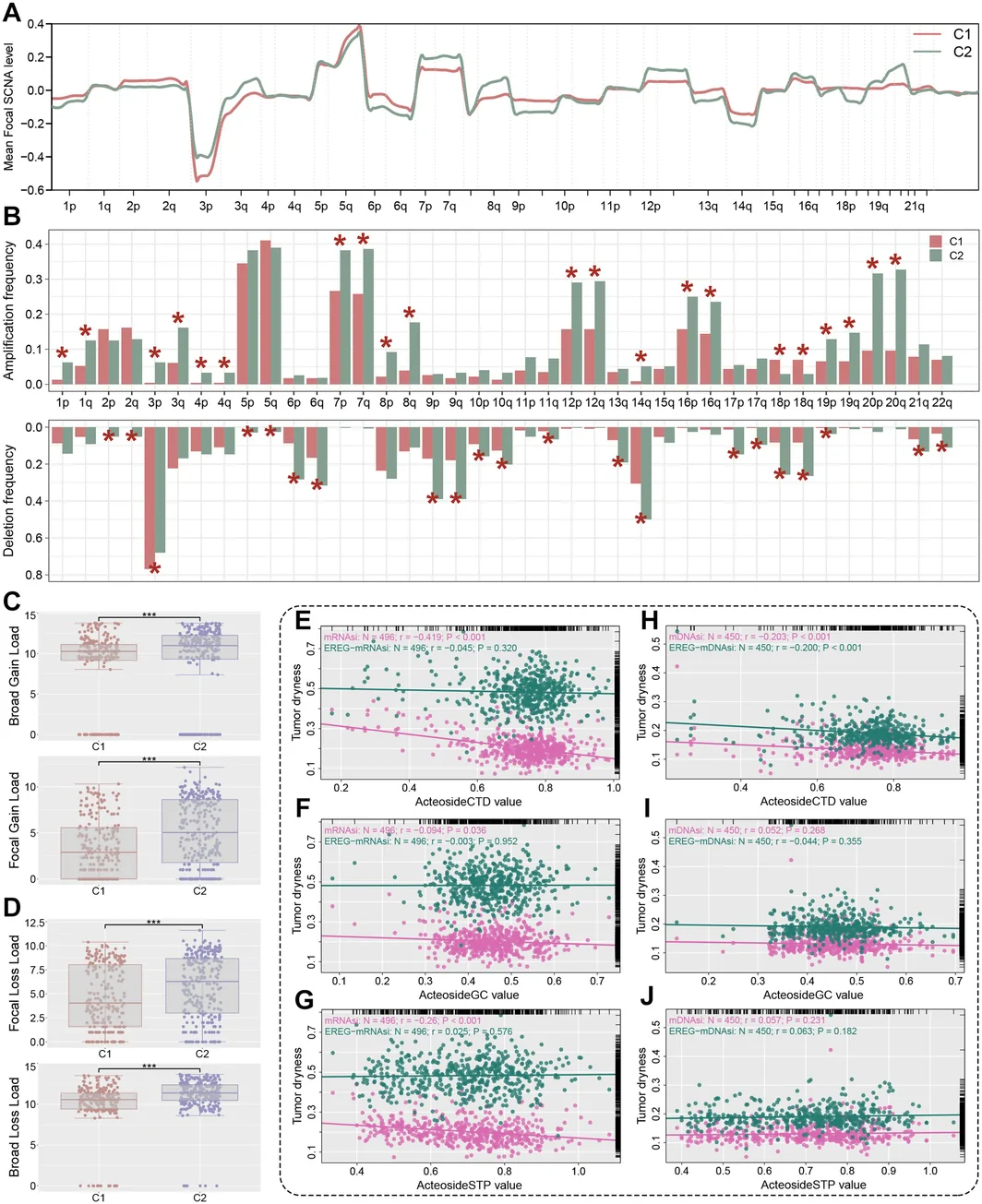
Genomic instability and tumor stemness profiles across acteoside‐related subtypes. (A, B) Somatic copy number alteration (SCNA) profiles in C1 and C2, showing (A) mean focal SCNA levels and (B) chromosomal amplification and deletion frequencies across chromosome arms (e.g., 7q, 12q, and 20p/q). (C, D) Comparison of (C) Broad and (D) Focal Gain/Loss Loads between subtypes using the Wilcoxon rank‐sum test (*p* < 0.001). (E–G) Pearson correlation between acteoside‐related scores (CTD, GC, and STP) and the transcriptome‐derived stemness index (mRNAsi). (H–J) Correlation between acteoside‐related scores and methylation‐derived indices (mDNAsi and EREG‐mDNAsi).

To examine the genetic basis of the subtypes, we analyzed their SCNA profiles. C2 showed more frequent chromosomal aberrations, including increased amplifications in 7q, 12q, 16p/q, and 20p/q and deletions in 9p and 14q (Figure 4A,B). Quantitative analysis confirmed higher Broad Gain/Loss and Focal Gain/Loss Loads in C2 than in C1 (*p* < 0.001), consistent with greater genomic instability in the high‐risk subtype (Figure 4C,D). Correlation analysis showed that all three acteoside‐related scores (CTD, GC, and STP) were negatively associated with the transcriptomic stemness index (mRNAsi) (Figure 4E–G). A similar negative correlation was observed between the ActeosideCTD score and the methylation‐based stemness index (mDNAsi) (Figure 4H–J). These results suggest that higher acteoside‐related signature activity is associated with lower tumor stemness and may partly explain the more favorable outcome of the C1 subtype.

### 3.5 Distinct Somatic Mutation Landscapes and Key Genomic Drivers

**FIGURE 5:**
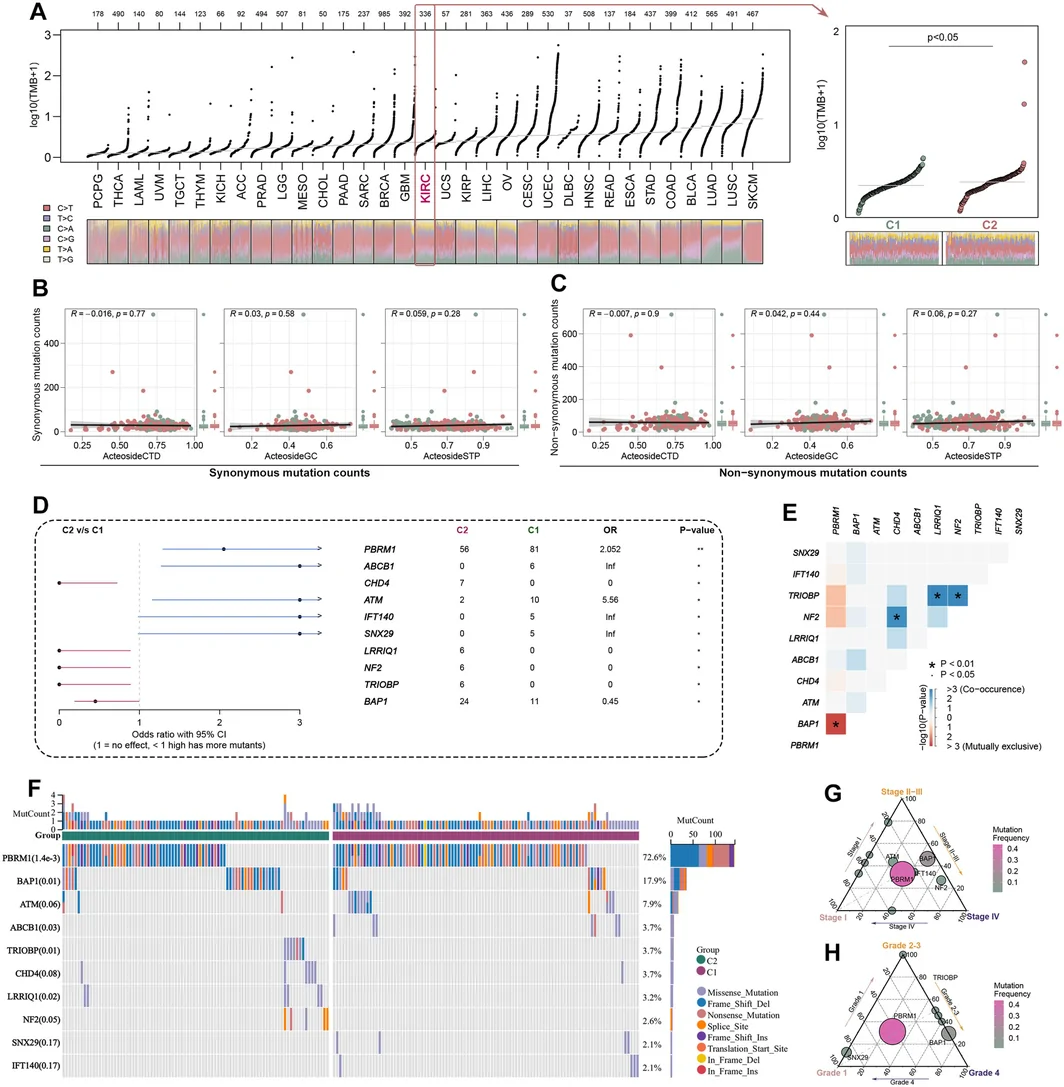
Somatic mutation profiles and genomic drivers of acteoside‐related subtypes. (A) Pan‐cancer TMB distribution (left) and KIRC‐specific comparison between C1 and C2 (right) using the Wilcoxon rank‐sum test (*p* < 0.05). (B, C) Pearson correlation between acteoside‐related scores and (B) synonymous and (C) non‐synonymous mutation counts. (D) Forest plot of differential mutation frequencies between C2 and C1, showing odds ratios (ORs) for PBRM1 and BAP1. (E) Heatmap showing mutual exclusivity (red asterisks) and co‐occurrence among high‐frequency mutations. (F) OncoPrint waterfall plot displaying the mutation landscape and frequencies of the top 10 genes across subtypes. (G, H) Ternary plots showing the distribution of key mutations across (G) clinical stages and (H) histological grades.

In the TCGA‐KIRC cohort, C2 had a higher TMB than C1 (*p* < 0.05), although acteoside‐related scores were not linearly correlated with total mutation counts (Figure 5A–C). Differential mutation analysis revealed subtype‐specific genomic patterns: PBRM1 mutations were more frequent in C1 (OR = 2.052, *p* < 0.01), whereas BAP1 mutations were enriched in C2 (OR = 0.45, *p* < 0.05) (Figure 5D). PBRM1 and BAP1 alterations were also mutually exclusive (*p* < 0.05) and showed different clinical associations (Figure 5E). According to the waterfall plot in Figure 5F, PBRM1 was the most frequently altered gene, whereas C2 showed a relatively higher proportion of BAP1 and ATM alterations. Ternary plot analysis showed that BAP1 mutations were enriched in advanced‐stage and high‐grade tumors, while PBRM1 mutations occurred more frequently in early‐stage tumors (Figure 5G,H). These findings link the poor prognosis of C2 to a BAP1‐associated genomic program.

### 3.6 Development and Multicenter Validation of the Integrated Prognostic Signature

**FIGURE 6:**
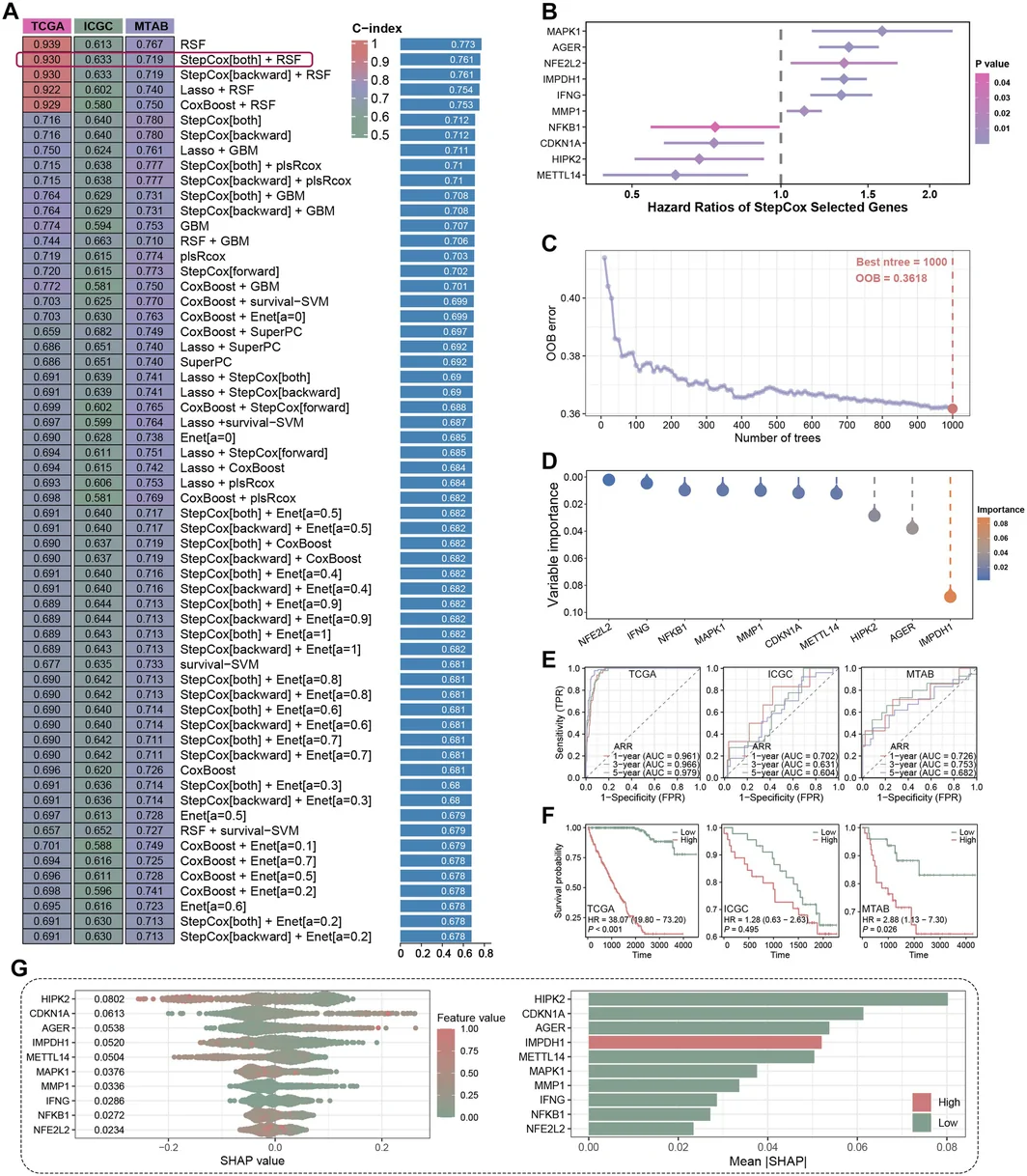
Development, validation, and interpretability of the integrated prognostic signature. (A) Benchmarking of 101 machine‐learning algorithm combinations across TCGA, ICGC, and E‐MTAB‐1980 cohorts; The StepCox [both] + RSF ensemble achieved the highest C‐index. (B–D) Identification of the 10‐gene signature, including (B) univariate Cox hazard ratios, (C) RSF model optimization based on OOB error, and (D) variable‐importance ranking. (E–F) Multicenter performance evaluation by (E) time‐dependent ROC curves and (F) Kaplan–Meier survival analysis for high‐ and low‐risk groups (*p* < 0.05). (G) SHAP‐based model interpretation showing feature contributions to risk prediction, with IMPDH1 as the main high‐risk contributor.

A prognostic model was constructed by benchmarking 101 algorithm combinations using the 29 genes significant in univariate Cox regression. The StepCox [both] + RSF ensemble achieved the highest C‐index across the TCGA (0.930), ICGC (0.633), and E‐MTAB‐1980 (0.719) cohorts (Figure 6A). This model yielded a 10‐gene signature, in which IMPDH1 had the highest variable importance in the RSF model (Figure 6B–D). The signature showed good predictive performance, with 5‐year AUCs of 0.979, 0.682, and 0.753 in the three cohorts, respectively (Figure 6E). Kaplan–Meier analysis showed worse overall survival in the high‐risk group across all datasets (Figure 6F). Calibration curves in the TCGA, ICGC, and E‐MTAB‐1980 cohorts showed acceptable agreement between predicted and observed survival probabilities (Figure S1A–C), and DCA indicated potential clinical net benefit of the risk score across a range of threshold probabilities (Figure S1D). A nomogram incorporating the prognostic score was also constructed for individualized survival prediction (Figure S1E). SHAP analysis identified IMPDH1 as the main positive contributor to risk prediction in high‐risk patients (Figure 6G). In additional univariate and multivariate Cox regression analyses incorporating age, sex, pathological grade, pathological stage, T stage, M stage, and risk score, the risk score remained independently associated with overall survival after adjustment for clinicopathological variables (Figure S1F).

### 3.7 IMPDH1 Promotes Proliferation and Migration of ccRCC Cells In Vitro

**FIGURE 7:**
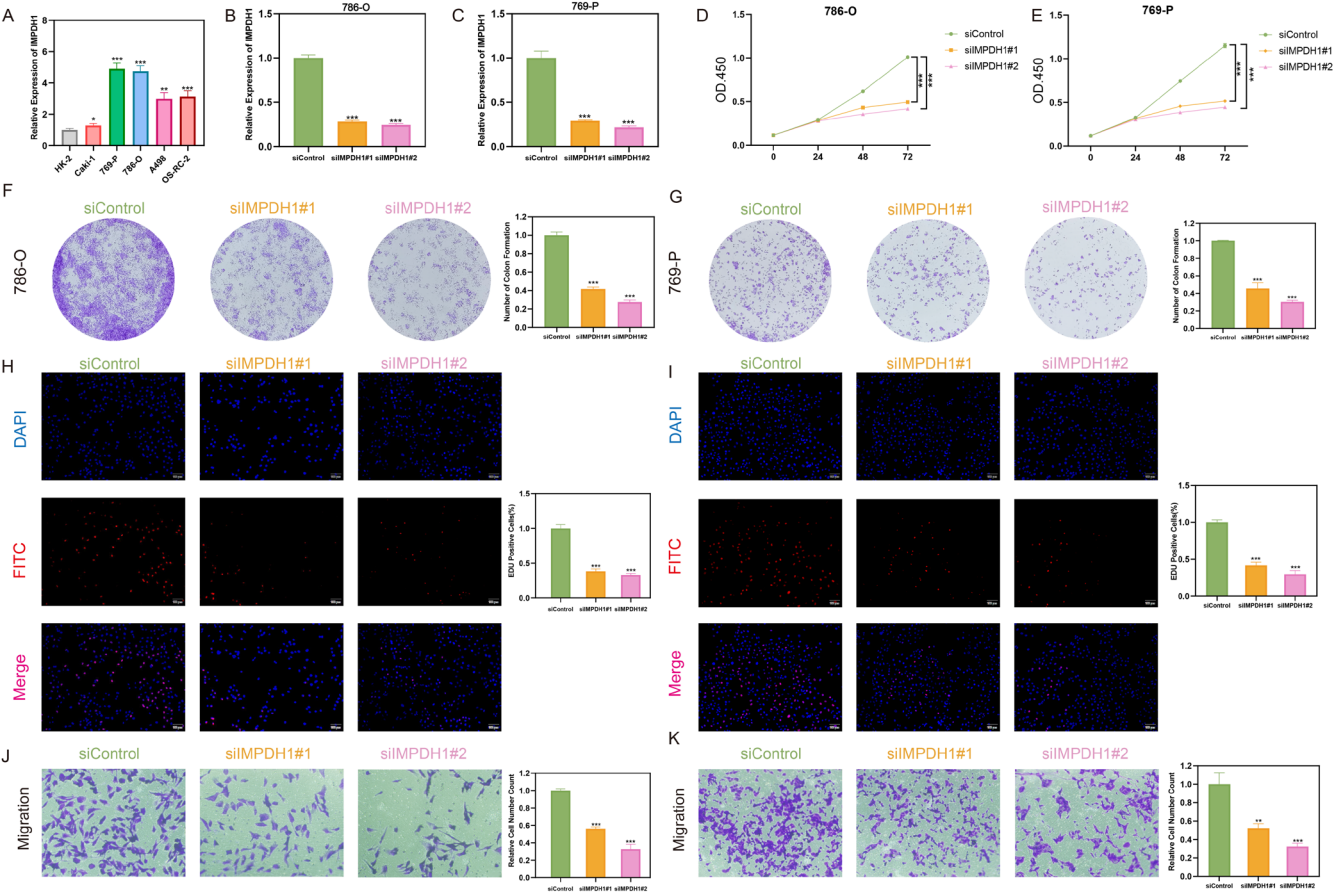
Silencing of IMPDH1 suppresses the malignant progression of ccRCC cells in vitro. (A–C) Validation of IMPDH1 knockdown efficiency in 786‐O and 769‐P cells using two independent siRNAs (siIMPDH1#1 and siIMPDH1#2) compared with siControl by qRT‐PCR. (D, E) CCK‐8 assays showing the effect of IMPDH1 silencing on proliferative viability in (D) 786‐O and (E) 769‐P cells over 72 h. (F, G) Representative images and quantification of colony formation assays in (F) 786‐O and (G) 769‐P cells after IMPDH1 knockdown. (H, I) EdU incorporation assays (scale bar = 100 μm) showing DNA synthesis in (H) 786‐O and (I) 769‐P cells; EdU‐positive cells (FITC, red) relative to total nuclei (DAPI, blue) were quantified. (J, K) Transwell migration images and quantification showing migratory capacity in (J) 786‐O and (K) 769‐P cells after IMPDH1 silencing.

**FIGURE 8:**
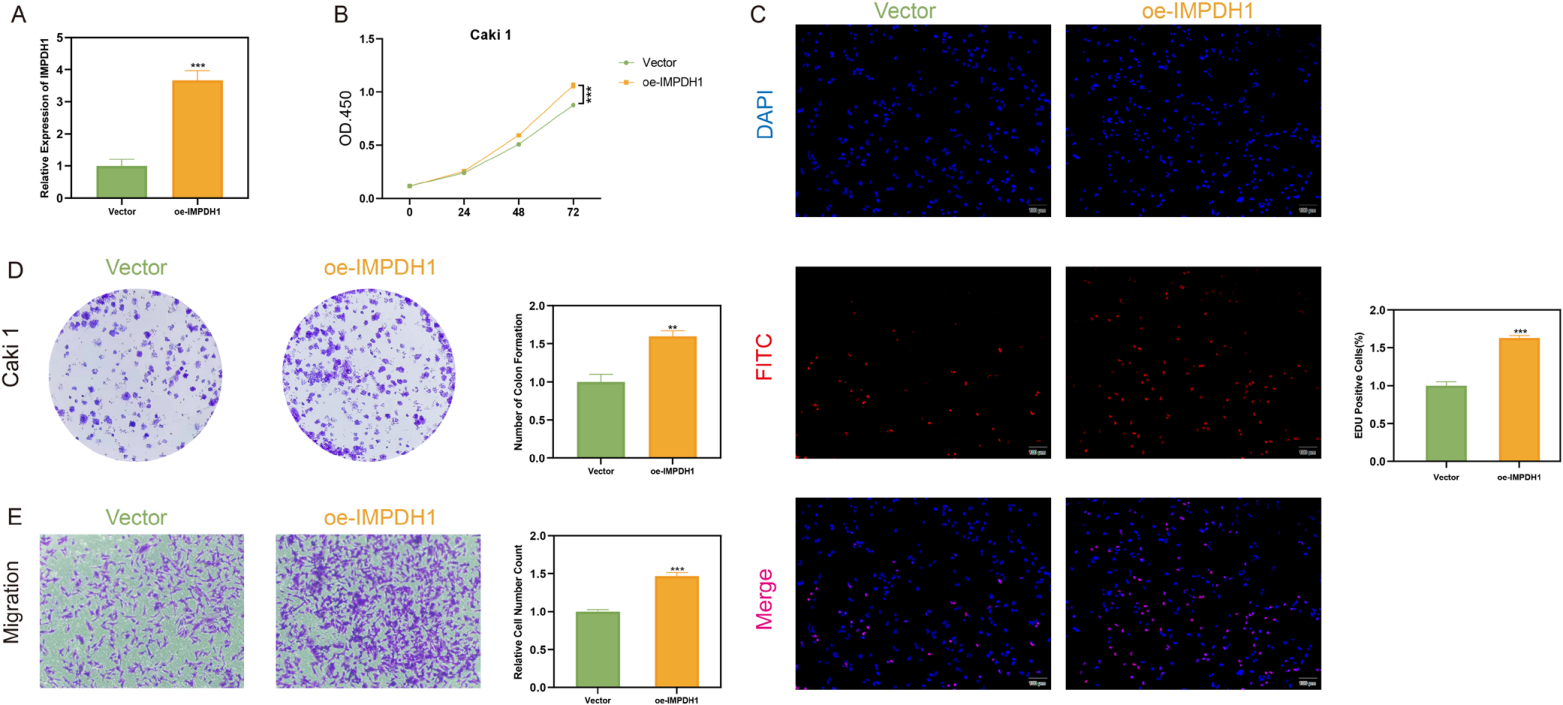
Overexpression of IMPDH1 promotes proliferation and migration in Caki‐1 cells. (A) qRT‐PCR analysis confirming IMPDH1 overexpression in Caki‐1 cells transfected with oe‐IMPDH1 relative to the empty vector. (B) CCK‐8 growth curves showing increased proliferation after IMPDH1 overexpression. (C) EdU incorporation assay (scale bar = 100 μm) and quantification of DNA synthesis in Caki‐1 cells transfected with vector or oe‐IMPDH1. (D) Representative images and statistical comparison of colony formation in control and IMPDH1‐overexpressing Caki‐1 cells. (E) Transwell migration images and quantification showing the effect of IMPDH1 overexpression on migratory capacity.

To validate the oncogenic role of IMPDH1, the top‐ranked prognostic driver in the machine‐learning signature, we performed loss‐ and gain‐of‐function assays in ccRCC cell lines. siRNA‐mediated IMPDH1 silencing in 786‐O and 769‐P cells was confirmed by qRT‐PCR, with GAPDH used as the internal reference gene and relative expression calculated using the 2^−ΔΔCt^ method (Figure 7A–C). CCK‐8 assays showed reduced proliferative viability after IMPDH1 knockdown compared with siControl cells (Figure 7D,E). Colony formation assays showed fewer and smaller colonies after IMPDH1 depletion (Figure 7F,G), and EdU assays showed a lower proportion of FITC‐positive cells, indicating reduced DNA synthesis (Figure 7H,I). Transwell assays further showed that IMPDH1 knockdown impaired ccRCC cell migration (Figure 7J,K). Conversely, IMPDH1 overexpression in Caki‐1 cells (Figure 8A) increased cell proliferation (Figure 8B), promoted EdU incorporation (Figure 8C), enhanced colony formation (Figure 8D), and increased migratory ability (Figure 8E). All cell experiments were performed with at least three independent biological replicates. Because the 24‐h Transwell assay may be partly influenced by altered proliferation, migration was interpreted together with the proliferation and EdU assays rather than as standalone evidence of metastatic potential. These results support a functional role for IMPDH1 in promoting proliferative and migratory phenotypes in ccRCC cells.

## 4 Discussion

ccRCC is characterized by metabolic reprogramming and genomic complexity, both of which are linked to relapse and drug resistance (Cotta et al. 2023; Hu et al. 2024). In this study, we connected predicted pharmacological targets of acteoside, a dietary polyphenol, with clinical and molecular features of ccRCC. Acteoside‐related enrichment scores were higher in malignant tissues and increased with histological grade and clinical stage, suggesting an association with tumor progression. Single‐cell and spatial transcriptomic analyses further localized these signatures to malignant compartments within the tumor microenvironment. These findings do not establish a preventive effect of acteoside in patients, but they provide a molecular basis for exploring how dietary polyphenol‐related pathways may intersect with ccRCC biology.

Two molecular subtypes, C1 and C2, were identified, with distinct biological programs and clinical outcomes. C1 was associated with better prognosis and showed an immune‐active and metabolically organized profile. In contrast, C2 represented a higher‐risk subtype with activation of DNA repair pathways and reduced enrichment of renal epithelial differentiation terms. These patterns suggest that acteoside‐related signatures capture a transition from a more differentiated metabolic state toward proliferation and genomic instability. The negative correlations between acteoside scores and tumor stemness indices (mRNAsi and mDNAsi) are consistent with this interpretation, although the mechanisms linking these signatures to differentiation or stemness remain to be defined.

The divergent outcomes of C1 and C2 were also reflected in their somatic mutation profiles, particularly the mutual exclusivity of PBRM1 and BAP1 alterations. PBRM1 mutations have been associated with relatively indolent disease biology and immune responsiveness and were enriched in C1. In contrast, BAP1 mutations, which are linked to high‐grade progression, metabolic dysfunction, and poor prognosis in ccRCC, occurred more frequently in C2. Together with higher TMB and greater SCNA burden, these findings provide a genomic basis for the aggressive features of C2 (Miao et al. 2018; Xia et al. 2026; Camp et al. 2026). They also suggest that acteoside‐related molecular subtypes may provide a basis for future investigation of subtype‐specific therapeutic strategies.

To translate the multi‐omics findings into an interpretable tool, we selected the StepCox + RSF ensemble from 101 machine‐learning algorithm combinations based on cross‐cohort performance. The resulting 10‐gene signature remained informative in the TCGA, ICGC, and E‐MTAB‐1980 cohorts, although its aim was not to replace clinicopathological staging systems. Instead, the model was used to stratify risk and to identify biologically relevant nodes linking acteoside‐related pharmacological signatures with ccRCC progression. IMPDH1 emerged as the most influential feature in the model according to variable‐importance and SHAP analyses. As a rate‐limiting enzyme in de novo guanine nucleotide biosynthesis, IMPDH1 supports the nucleotide demand of rapidly proliferating tumor cells (Zhang et al. 2023; Huang et al. 2018). Our in vitro experiments showed that IMPDH1 knockdown reduced proliferation, colony formation, DNA synthesis, and migration, whereas IMPDH1 overexpression produced the opposite effects. These data support IMPDH1 as both a prognostic marker and a candidate metabolic vulnerability in ccRCC.

From a food and nutritional perspective, the interpretation of these findings requires particular attention to the gastrointestinal fate of acteoside. Verbascoside occurs in edible and beverage‐related plant matrices, yet the amount present in a food cannot be assumed to correspond directly to the concentration reaching systemic tissues. Experimental studies indicate incomplete digestive recovery, limited intestinal uptake, and low systemic availability of intact acteoside, together with extensive microbial and host metabolism. Consequently, acteoside‐related molecular signatures identified from predicted target information should not be interpreted as biomarkers of dietary acteoside exposure. Rather, they define disease‐relevant host molecular programs that overlap with the pharmacological space associated with this food‐derived polyphenol. This distinction is important when translating molecular findings toward nutrition because the biologically relevant exposure after consumption may include the parent compound together with hydroxytyrosol, caffeic‐acid‐related products, hydroxyphenylpropionic acids, and other metabolites generated during digestion and microbial transformation.

The present findings may nevertheless contribute to food and nutrition research by identifying molecular endpoints that can be prioritized in subsequent dietary‐polyphenol studies. Acteoside‐related programs in our analysis were associated with immune activity, metabolic organization, renal epithelial differentiation, tumor stemness, genomic instability, and clinical outcome. Within this framework, IMPDH1 emerged as a major risk‐associated feature, and functional perturbation confirmed its role in ccRCC proliferation, DNA synthesis, clonogenic growth, and migration. These experiments do not demonstrate that IMPDH1 is directly regulated or bound by acteoside. Instead, they identify guanine nucleotide metabolism as one candidate host pathway at the intersection of acteoside‐related molecular biology and ccRCC progression. Future food‐science and nutrition studies can therefore test whether this pathway, together with the broader molecular signatures identified here, is modifiable by acteoside‐containing food matrices, purified acteoside, or gastrointestinal metabolites at physiologically achievable exposure levels. Such studies would ideally integrate quantitative characterization of food matrices, simulated gastrointestinal digestion, metabolite profiling, intestinal transport models, and ultimately controlled dietary intervention.

Several limitations should be noted. First, although our analysis links acteoside‐related gene sets to ccRCC progression, direct biochemical interactions between acteoside and predicted molecular targets, including IMPDH1, remain to be confirmed using appropriate target‐engagement assays. Second, the present study did not quantify dietary acteoside intake, measure circulating or tumor concentrations of acteoside or its metabolites, or directly expose ccRCC cells to acteoside or its gastrointestinal metabolites. Accordingly, the molecular signatures identified here should be interpreted as hypotheses derived from acteoside‐related predicted target space rather than evidence of target engagement following dietary intake. Third, although the prognostic model was validated in multiple independent cohorts, these datasets were retrospective; prospective validation will be needed before clinical application. Fourth, the spatial transcriptomic analysis was based on two available ccRCC sections, limiting statistical generalization across the full spectrum of intratumoral spatial heterogeneity. Future studies should integrate quantitative food‐matrix characterization, gastrointestinal digestion and microbial metabolism, physiologically relevant exposure experiments, and controlled dietary intervention to determine whether the molecular nodes identified here can actually be modulated through nutritional exposure.

In summary, this study maps acteoside‐related molecular features in ccRCC by integrating bulk, single‐cell, and spatial transcriptomic data with machine‐learning‐based prognostic modeling and functional validation. The identification of IMPDH1 highlights a disease‐relevant metabolic node within the acteoside‐associated molecular landscape. From a food and nutritional perspective, these findings provide a molecular prioritization framework rather than evidence that dietary acteoside itself prevents ccRCC. Future studies incorporating food source, gastrointestinal bioaccessibility and metabolism, physiologically relevant parent‐compound and metabolite exposure, and controlled dietary intervention will be necessary to determine whether the molecular programs identified here are nutritionally modifiable.

## Author Contributions

**Xu Chen:** funding acquisition, conceptualization, investigation, methodology, validation, visualization, writing – review and editing, writing – original draft, project administration, formal analysis, software, data curation, supervision, resources. **Dong Zhang:** conceptualization, methodology, software, data curation, formal analysis, validation, visualization, resources, investigation. **Ziyu Ji:** validation, visualization, methodology, conceptualization. **Shuxian Sun:** investigation, validation, formal analysis, supervision. **Jia Wang:** investigation, conceptualization, funding acquisition, visualization. **Shuijie Shen:** funding acquisition, conceptualization, investigation, writing – original draft, writing – review and editing, project administration. **Jian Zhu:** writing – original draft, funding acquisition, investigation, conceptualization, methodology, validation, visualization, writing – review and editing, project administration, formal analysis, software, data curation, supervision, resources.

## Funding

This work was supported by the Nantong University Special Research Fund for Clinical Medicine (Grant 2025JY044) and the Scientific Research Project of Jiangsu Association of Chinese Medicine (Grant ZXFZ2024097).

## Ethics Statement

The authors have nothing to report.

## Consent

The authors have nothing to report.

## Conflicts of Interest

The authors declare no conflicts of interest.

## Supporting information


**Figure S1:** Supplementary evaluation of the prognostic signature. (A–C) Calibration curves for 1‐, 3‐, and 5‐year survival (TCGA, ICGC, E‐MTAB‐1980). (D) Decision curve analysis. (E) Nomogram. (F) Univariate and multivariate Cox regression (TCGA, age, sex, grade, stage, T, M, risk score).

**Table S1:** Lists of acteoside‐related target genes obtained from the CTD, GeneCards, and SwissTargetPrediction databases.

## Data Availability

The data that support the findings of this study are available from the corresponding author upon reasonable request.
